# Integrins in Trabecular Meshwork and Optic Nerve Head: Possible Association with the Pathogenesis of Glaucoma

**DOI:** 10.1155/2013/202905

**Published:** 2013-03-18

**Authors:** Yisheng Zhong, Jing Wang, Xunda Luo

**Affiliations:** ^1^Department of Ophthalmology, Ruijin Hospital Affiliated Medical School, Shanghai Jiaotong University School of Medicine, 197 Rui Jin Er Road, Shanghai 200025, China; ^2^Scheie Eye Institute, Department of Ophthalmology, University of Pennsylvania, Philadelphia, PA 19104, USA

## Abstract

Integrins are a family of membrane-spanning proteins that are important receptors for cell adhesion to extracellular matrix proteins. They also provide connections between the extracellular environment and intracellular cytoskeletons and are responsible for activation of many intracellular signaling pathways. In vitro and in vivo data strongly indicate that integrin-mediated signaling events can modulate the organization of the actin cytoskeleton in trabecular meshwork (TM) cells and are associated with astrocyte migration and microglia activation of the optic nerve head in patients with primary open angle glaucoma. Consequently, increase in resistance in the TM outflow pathways and remodeling of the optic nerve head occur, which in turn increases intraocular pressure (IOP), adds additional mechanical stress and strain to optic nerve axons, and accelerates damage of axons initially caused by optic nerve head remodeling. Integrins appear to be ideal candidates for translating physical stress and strain into cellular responses known to occur in glaucomatous optic neuropathy.

## 1. Introduction

Glaucoma is the second leading cause of blindness worldwide. It is estimated that there are 60.5 million people with glaucoma worldwide in 2010 and will increase to 79.6 million by 2020 [1]. Glaucoma is commonly defined as an optic neuropathy that is associated with characteristic structural damage to the optic nerve and associated with visual dysfunction that might be caused by various pathological processes [2–4]. Risk factors related to glaucoma include intraocular pressure (IOP), age, family history, clinical appearance of the optic nerve, race, and potential vascular disease [5–8]. IOP remains the most prominent risk factor of all, and lowering IOP is the mainstay of nearly all contemporary glaucoma therapies.

Several prospective randomized multicenter studies have identified that IOP reduction with either medication or surgery can lower the incidence of the disease and slow down progression of vision loss in glaucoma patients [9–15]. If IOP is beyond the tolerable range of the optic nerve, retinal ganglion cells (RGCs) axons degenerate at the optic nerve head in the region of the lamina cribrosa, a process that occurs in parallel to the apoptotic death of retinal ganglion cells (RGCs). The exact mechanisms that lead to the death of RGCs in glaucoma have not been fully identified but might include blockade of both anterograde and retrograde axonal transport which lead to deprivation of neurotrophic signals [4]. Glaucomatous neuropathy might occur in parallel to extracellular matrix (ECM) remodeling of the optic nerve head [4, 16, 17].

IOP is generated in the anterior eye via the aqueous humor circulation system. The trabecular meshwork (TM) outflow pathway offers a certain resistance to aqueous humor outflow, and, in response to the resistance, IOP is generated [18, 19]. In addition, there is an alternative uveoscleral pathway along the interstitial spaces of the ciliary muscle and the supraciliary space, of which physiological role is not fully understood [20]. Much evidence indicates that normal aqueous humor outflow resistance mainly resides in the inner wall region of the TM outflow pathways [18, 21]. However, the exact structure and molecular nature of trabecular outflow resistance in the inner wall region are not clear. Two hypotheses have been proposed for the trabecular outflow resistance [22]. One hypothesis is based on the observation that banded fibrillar ECM in the juxtacanalicular region of the TM in glaucomatous eyes is significantly thicker than that seen in normal eyes [23, 24], which leads to the assumption that the elevation of outflow resistance is attributable to changes in the quantity and property of the ECM in this region [25, 26]. The ECM hypothesis is supported by the observation that perfusion of anterior eye segments in organ cultures with metalloproteinases that digest ECM components leads to a reversible increase in outflow facility [27]. Another hypothesis is based on the discovery that the cells of TM have contractile properties [28] and that an increase in TM tone increases outflow resistance [29]. Thereby, an increase in the contraction state of TM cells in glaucoma might lead to higher rigidity of the TM and to an increased outflow resistance. The contractility hypothesis is supported by the observation that experimental disruption of the actin cytoskeleton of the TM decreases outflow resistance [30, 31] and by recent findings which provide evidence that the TM of patients with primary open angle glaucoma (POAG) is stiffer than that of age-matched controls [32]. The two hypotheses are not mutually exclusive, as it is possible that TM cells that increase their contractile capabilities simultaneously synthesize more fibrillar matrix to transmit more force.

Research efforts have been put in the last decade to elucidate cells response and ECM remodeling process in the TM and the optic nerve head in glaucoma, and integrins have been identified as very important participants in this process. The purpose of this paper is to summarize findings on integrins in pathogenesis of glaucoma.

## 2. The Integrins and Their Ligands

Integrins exist as heterodimers with *α* and *β* subunits that interact noncovalently. At present, eighteen *α*- and eight *β*- subunits have been identified and known to form 24 different heterodimers (Table 1), and an addition of six *α*-subunits and one *β*-subunit is now speculated, based upon analysis of the human genome [33–35]. Integrins are a family of membrane-spanning proteins that are important receptors for cell adhesion to ECM proteins. They also provide connections between the extracellular environment and intracellular cytoskeletons. In addition to their roles with ECM, they are now known to be responsible for activation of many intracellular signaling pathways to mediate many cell-cell interactions including those involved in inflammation, hemostasis, and cancer metastasis and to serve as cell receptors for viruses and bacteria [34].

Each integrin subunit appears to have one or more ligand to which it can bind. Specificity of ligand recognition for a heterodimer is influenced by the specific combination of subunits and relative affinity and availability of the ligands [36]. In general, integrin ligands can be classified into four categories: ECM, soluble molecules, cell-cell, and pathogens and toxins [33]. Ligands of integrins are listed in Table 1 [36, 37]. Integrins can be divided into four major types based on ligand sequences that they recognize, which are arginine-glycine-aspartate (RGD) receptors, collagen receptor, laminin receptor and leukocyte-specific receptors [37] (Table 1).

## 3. Integrins in TM

TM cell-matrix adhesion is crucial for the maintenance of the aqueous humor outflow resistance. ECM components in TM include fibronectin, laminin, vitronectin, types I, III, IV, V, and VI collagen, fibrillin-1, decorin, and others (Table 2) [38–49]. Fibronectin, vitronectin, and collagen types I and IV are the major extracellular matrix proteins in human TM [50] and are ligands of integrins as well. Many biological activities of ECM in TM are mediated via integrin-ECM interactions [51]. It has been identified that steroid significantly increases fibronectin expression in TM cells [52, 53]. Tissue/organ culture experiments suggest that overexpression of ECM components (laminin and collagen type IV, etc.) by TM cells may play a role in the development of outflow resistance and contribute to the development of steroid-induced glaucoma and POAG [24, 54, 55].

Integrins play a critical role in TM cell-matrix adhesion. In TM cell culture, attachment of cells to ECM proteins can be blocked by specific integrin antibodies, and cell adhesion to fibronectin and vitronectin can also be inhibited by peptides containing Arg-Gly-Asp (RGD) sequences [38]. Up to date, there are thirteen integrin subunits known to be expressed by TM cells (Table 2) [38, 39, 43], and it is possible that more subunits might be found in the future. These integrins are distributed throughout the TM with the peak expression observed along cells on the beams, and the expression does not appear to vary with donor age [39]. In cultured human TM cell lines, expressions of *α*2, *α*5, and *α*v integrin subunits changed consistently when dexamethasone is used, and there was no difference in expression patterns of any of these integrin subunits between cell lines obtained from normal and glaucomatous eyes [56]. In addition, connective tissue growth factor can also mediate upregulation of integrin subunits *α*v and *β*1 expressions in TM cells [57].

Integrins form important physical links between extracellular environment and intracellular actin cytoskeleton and may provide a mechanism to detect changes in external forces in the microenvironment of the TM [58]. Cross-linked actin networks (CLANs) are originally observed in spreading cells and are described as actin geodesic domes. CLANs are composed of interconnected arrays of three-to-five actin filaments extending outward from a central vertex. They may be precursors to actin stress fibers that regulate contractility in cells. Studies in cultured anterior segments and cultured TM cells treated with dexamethasone have suggested that steroid can lead to rearrangement of actin cytoskeleton into CLANs that resemble geodesic domes or polygonal actin networks [59–64]. CLANs have also been observed in cultured TM cells and in TM cells in isolated meshworks from glaucomatous donor eyes in the absence of any dexamethasone treatment [65, 66]. CLANs have also been found in normal TM cells in isolated meshworks, albeit at a lower frequency than in glaucomatous TM [66, 67]. Recently, it has been suggested that CLANs formation in TM cells may reduce contractility of the tissue by increasing the rigidity of the cells and thus rendering them unable to change shape and “relax” under pressure. Alternatively, CLANs formation could impact other actin-mediated biological processes of the TM that are required for normal outflow facility such as attachment to the ECM, phagocytosis, and gene expression [68–70], which suggests that these actin structures could possibly be involved in pathogenesis of steroid-induced glaucoma and other forms of POAG [62, 63, 68, 70]. CLANs formation can be regulated by *β*1 and *α*v*β*3 integrin signaling pathways. Distinct *β*1 and *α*v*β*3 integrin signaling pathways converge to enhance CLANs formation [42]. *β*1-mediated CLAN formation is PI-3 K dependent, whereas *β*3-mediated CLAN formation is CD47 and Rac1/Trio-dependent and might be regulated by thrombospondin-1. Both integrin pathways are Src dependent [58, 71]. Therefore, integrin-mediated signaling events can modulate the organization of the actin cytoskeleton in TM cells and consequently participate in regulation of cytoskeletal events previously demonstrated to be involved in regulation of outflow facility [42, 51].

It has been found that the active site in the heparin II (HepII) domain of fibronectin could regulate outflow facility in cultured anterior segment and disrupt actin cytoskeleton in transformed human TM (TM-1) cells, and the active site in the HepII domain is the syndecan/integrin binding sequence, PPRARI [72]. The PPRARI sequence in the HepII domain has been shown to serve as a physiological *α*4*β*1 ligand [73], and soluble anti-*α*4 integrin antibodies could inhibit Hep II domain-mediated cell spreading and soluble vascular cell adhesion molecule-1- (*α*4*β*1-ligand) induced cell spreading, which suggests the Hep II domain mediates cell spreading and stress fiber formation through *α*4*β*1 integrin, a potentially key regulator of tissue contractility [74]. Recently, it has been reported that *β*1 integrin function-blocking antibody inhibits adhesion and spread of TM cells on Galectin-8- (Gal8-) coated wells. Phosphorylated myosin light chain 2 (MLC2) accumulates in cells adhered to Gal8 and is associated with stress fiber formation that can be abolished by Rho inhibitor, C3 transferase, or Rho-kinase (ROCK) inhibitor Y27632. These findings suggest that *β*1 integrins and the Rho/ROCK/MLC2 signaling pathway may be involved in Gal8-promoted cytoskeletal rearrangement in TM cells [75].

## 4. Integrins in Schlemm's Canal

It has been found that collagen I, IV, and laminin-511 are the prominent structural proteins in Schlemm's canal (SC) basement membrane [76], and SC cells express *α*2, *α*3, *α*6, *β*1, and *β*4 integrin subunits, and *α*6 is uniquely expressed by SC cells in situ in the conventional outflow tract and in vitro by cultured SC cells [76, 77]. The integrin-mediated SC cell-matrix adhesion may have a critical role in maintaining a continuous barrier to fluid flow.

## 5. Integrins in the Optic Nerve Head

Known components of the ECM in the optic nerve head are listed in Table 2 [78–97]. It is possible that more new components of ECM in optic nerve head might be found in the future. The major ECM proteins of optic nerve head consist of collagen types I, III, V, and VI, along with elastin in the peripapillary sclera, cores of laminar beams, and retrobulbar optic nerve septae. The basement membrane components laminin and collagen type IV are identified along the margins of the laminar beams in association with astrocytes and within the beams in association with capillary vascular endothelium [82, 83]. In addition to this, chondroitin sulfate- and dermatan sulfate-containing proteoglycans have also been identified in the primate optic nerve head [84]. Chondroitin and dermatan sulfate are localized to the lamina cribrosa and peripapillary sclera tissues that are load-bearing structures of the optic nerve head and more likely to be exposed to IOP [86]. 

Multiple integrin subunits have been found in the normal optic nerve head (Table 2). In normal eyes, *α*2, *α*3, *α*6, *β*1, and *β*4 integrin subunits are localized in astrocytes along the margins of laminar beams and within glial columns [16, 86], which suggests that integrins *α*2*β*1, *α*3*β*1, *α*6*β*1, and *α*6*β*4 may participate in attachment of astrocytes to basement membranes via laminin and sense changes in stress and strain within and anterior to the lamina cribrosa. *α*3, *α*5, *α*6, *α*v, *β*1, and *β*4 integrin subunits are localized in vascular endothelial cells [86, 98], which suggests that vascular endothelial cell response to stress may be mediated by integrins *α*3*β*1, *α*6*β*1, and *α*6*β*4, along with *α*5*β*1 and *α*v*β*1. *α*1, *β*2, and *β*3 integrin subunits are rarely expressed in any of the structures of the optic nerve head with a possible exception of *β*3 in larger blood vessel walls [86]. In glaucomatous optic nerve head, cells anterior to the compressed lamina cribrosa show persistent expression for *α*2, *α*3, *β*1, and *β*4 integrin subunits, whereas the expression for *α*4 subunit increases and the expression for *α*6 subunit decreases [86]. In eyes with advanced glaucoma damage, reduced *α*6 expression and variable expression for *β*4 anterior to the lamina cribrosa suggest astrocyte migration, and increased *α*4 subunit expression suggests microglia activation [86]. The heavy expression of integrins in association with astrocytes migration and microglia activation of the optic nerve head clearly suggests that these integrins can play an important role in glaucomatous neuropathy.

## 6. Concluding Remarks

In vitro and in vivo data strongly indicate that integrin-mediated signaling events can modulate the organization of actin cytoskeleton in TM cells and are associated with astrocytes migration and microglia activation in the optic nerve head of glaucoma patients. As a result, increase in resistance in the TM outflow pathways and ECM remodeling of the optic nerve head occur. While increase in outflow resistance causes an increase in IOP, and the remodeling of the optic nerve head accompanies the optic nerve axons damage. Increase in IOP further adds mechanical stress and strain to optic nerve axons and accelerates axon damages. Integrins appear to be ideal candidates for translating physical stress and strain into cellular responses known to occur in glaucomatous optic neuropathy.

## Figures and Tables

**Table 1:** Human integrin subunits and their ligands.

Integrin	Ligand	Type of receptors
*α*5*β*1	Fibronectin	RGD receptors
*α*8*β*1	Fibronectin/vitronectin/nephronectin	
*α*v*β*1	Fibronectin/vitronectin	
*α*v*β*3	Vitronectin/fibronectin/fibrinogen	
*α*v*β*5	Vitronectin	
*α*v*β*6	Fibronectin	
*α*v*β*8	Vitronectin	
*α*IIb*β*3	Fibrinogen/fibronectin	

*α*1*β*1	Collagen I/collagen IV/collagen IX	Collagen receptor
*α*2*β*1	Collagen I/collagen IV/collagen IX	
*α*10*β*1	Collagen II/collagen IV/collagen VI/collagen IX	
*α*11*β*1	Collagen I/collagen IV/collagen IX	

*α*3*β*1	Laminin-511/laminin-332/laminin-211	Laminin receptor
*α*6*β*1	Laminin-511/laminin-332/laminin-111/laminin-411	
*α*6*β*4	Laminin-511/laminin-332	
*α*7*β*1	Laminin-511/laminin-211/laminin-111/laminin-411	

*α*4*β*1	Fibronectin/VCAM-1	Leukocyte-specific receptors
*α*4*β*7 *α*9*β*1	MadCAM-1/fibronectin/VCAM-1 tenascin-C/VEGF-C/VEGF-D	
*α*D*β*2	ICAM-3/VCAM-1	
*α*L*β*2	ICAM-1/ICAM-2/ICAM-3/ICAM-5	
*α*M*β*2	iC3b/fibronectin + more	
*α*X*β*2	iC3b/fibronectin + more	
*α*E*β*7	E-cadherin	

RGD: arginine-glycine-aspartate, MadCAM: mucosal addressin cell adhesion molecule, ICAM: intercellular adhesion molecule, VCAM: vascular celladhesion molecule, and VEGF: vascular endothelial growth factor.

**Table 2:** The extracellular matrix and integrins in trabecular meshwork and optic nerve head.

Extracellular matrix	Integrin subunits
Trabecular meshwork	

Fibronectin	*α* l
Laminin	*α*2
Vitronectin	*α*3
Collagen type I	*α*4
Collagen type III	*α*5
Collagen type IV	*α*6
Collagen type V	*α*v
Collagen type VI	*β*1
Collagen type XII	*β*3
Fibrillin-1	*β*4
Decorin	*β*5
Elastin	*β*6
Cochlin	*β*7
Thrombospondin-1	
VCAM-1	
Myocilin	
Neuropilin-1	
Cadherin	
*β*-catenin	
Sphingosine 1	
Chondroitin sulfate	
Heparan sulfate	
Tenascin	

Optic nerve head	

Collagen type I	*α*l (little expression)
Collagen type III	*α*2
Collagen type IV	*α*3
Collagen type V	*α*4
Collagen type VI	*α*5
Collagens type VIII	*α*6
Collagens type IX	*α*v
Collagen type XVIII	*β*1
Laminin	*β*2 (little expression)
Fibronectin	*β*3 (little expression)
Tenascin	*β*4
Vitronectin	
Elastin	
Chondroitin sulfate	
Dermatan sulfate	
Aggrecan	
Entactin/nidogen	
Thrombospondin	
Thrombomodulin	
Endostatin	
Cadherin	
Periostin	
Cartilage linking protein-1	
Fibulin 1	
Decorin	
Perlecan	
Biglycan	
Versican	
Fibromodulin	
